# Analyzing the situation of older family caregivers with a focus on health-related quality of life and pain: a cross-sectional cohort study

**DOI:** 10.1186/s12955-020-01321-3

**Published:** 2020-03-20

**Authors:** Cecilia Fagerström, Sölve Elmståhl, Lena Sandin Wranker

**Affiliations:** 1 Blekinge Center of Competence, Karlskrona, Sweden; 2 Department of Health and Caring Science, Linnaeus University, Kalmar, Sweden; 3 Division of Geriatric Medicine, Department of Clinical Sciences, Lund University, Lund, Sweden; 4 Division of Geriatric Medicine, Department of Health Sciences, Lund University, Lund, Sweden

**Keywords:** Being worried, Family caregivers, Health-related quality of life, Older people, Pain

## Abstract

**Background:**

For a significant proportion of the older population, increasing age is associated with health problems and worsening health. Older family caregivers are largely responsible for care of next-of-kin living at home, which impacts their own physical and mental health both positively and negatively. However, evidence is insufficient regarding the health situation of older caregivers. The aim of this study was to investigate health-related quality of life (HRQoL) and pain, and their associations, among caregivers aged ≥60 years.

**Methods:**

The participants (*n* = 3444) were recruited from the Swedish National Study on Aging and Care-Blekinge and Good Aging in Skåne during 2001–2004. Participants aged ≥60 years were selected randomly and underwent cognitive tests, with demographic information obtained through questionnaires. The response rate was 60%. A predefined research protocol was used. HRQoL was measured with the Short-Form Health Survey, dimension mental health. Logistic regression models were used to investigate the associations between HRQoL and pain as well as control factors.

**Results:**

Family caregiving was reported by 395 (11.5%) of the participants, and 56.7% of the caregivers reported pain. Family caregivers reported lower pain intensity on the Visual Analogue Scale and were younger, on median, than non-caregivers. Irrespective of caregiver status, pain was associated with mental HRQoL. Concerns about personal health and financial status had the strongest associations with mental HRQOL in both groups, but the levels were higher among caregivers.

**Conclusion:**

Pain was one factor associated with low HRQoL regardless of family caregiver status and remained important when controlling for factors related to advanced age. This finding remained among family caregivers, though they reported lower pain intensity. Factors other than pain were shown to be important to mental HRQoL and should also be taken into consideration when discussing actions for family caregivers to maintain and improve health and HRQoL.

**Trial registration number:**

Not applicable.

## Introduction

For a significant proportion of the older population, especially those over 80 years, increasing age is associated with health problems. Already at over 60 years of age, half of the population reports pain [1], and this proportion increases with age [2]. Various disabilities, including pain, impact negatively on functional ability and quality of life (QoL) [3, 4]. Most members of this population live in their own homes and are integrated in society, but are in some way dependent on care and health services. The challenges of caring for the aging population are increasing, as are the demands on informal and formal caregivers [5]. Regardless of the context in which care and services for older people with disabilities are arranged, the importance of informal carers is substantial [6]. Care for older people would probably not be possible without the contributions of family members. The person who most often takes on the role as caregiver is a spouse [7] – usually an elderly person, who may have reduced health, pain, and impaired QoL him−/herself. The current situation of the older family caregivers, with attendant risks of increased functional, health- and age-related problems, combined with the rising numbers of family caregivers, deserves attention. Although the impact of caregiving on QoL among older people has been observed in the literature, evidence remains insufficient as regards older family caregivers’ health-related QoL (HRQoL) and its relation to pain.

A family caregiver, also called an informal caregiver, can be defined as a person who helps a significant other or another person in the family with personal and instrumental activities in daily life (ADL). Bowers [8] identified five levels of caregiving: anticipatory care (based on future needs), preventive care (encompassing both physical and mental illnesses), supervisory care (for example arranging doctors’ appointments and checking up on the person), instrumental and personal care (physical needs), and, lastly, protecting the self-esteem of the person cared for. Therefore, a family care provider activity must be interpreted and evaluated in its specific context, as the concept of caregiving is broad.

There is a lack of population-based studies on health and QoL among older family caregivers. When prior studies have highlighted the frequency of health problems among caregivers, they have often focused on informal caregivers of family members with dementia, and the caregivers’ QoL [9]. The role of a family caregiver and his/her increased burden [10] are well-described in the literature and usually diagnosis-based, in the context of dementia care. Compared with research on the impact of caregiving on psychological health, such as burden, research on the impact of caregiving on functional and physical health is still limited and the existing studies have produced more ambiguous results [11].

While QoL involves a broad multidimensional evaluation of the intra-personal and person-environmental system of an individual [12], and the individual’s perceptions of the aging process and self-identity, HRQoL focuses on how physical and mental health affects the day-to-day demands of life and whether the individual’s ability to fulfil needs and desires is constrained by health [13]. Within clinical healthcare, the focus has shifted to individuals’ HRQoL [14], in order to assess clinical outcomes and changes in individuals’ health status. The goal is to gather evidence on health to enable use or HRQoL as an outcome alternative to QoL, which has a multidimensional nature and is affected by a broad range of factors.

In western societies like Sweden, family caregivers account for most of the care delivered [15]. Many family caregivers feel they have no choice in becoming family caregivers [16]. Schultz et al. stressed that a lack of choice was connected to negative health impacts, physical strain, and high levels of stress [16]. In addition, Barrow and Harrison showed that informal care at home negatively influenced caregivers’ health, resulting in bodily pain, psychiatric morbidity, and obesity [17]. In their study, health tended to be worse when the caregivers had fewer friends and relations in their neighborhood [17]. Barrow and Harrison’s study did not discuss the caregivers’ HRQoL; knowledge about the relation between HRQoL and health problems is still lacking in this context.

Pain, defined by the International Association for the Study of Pain as an “unpleasant sensory and emotional experience associated with actual or potential tissue damage or described in terms of such damage” [18] (p. 475), is related to advanced age [17]. Many members of the older population suffer from pain in one or more body parts [1], regardless of caregiver status.

Unimpeded function can be expected to be particularly important for those who become family caregivers, as they are expected to give care, entailing certain duties and requirements, regardless of age and whether they have chosen to become caregivers.

However, physical health problems like pain might be present more often in caregivers due to caregiving activities and the stress and responsibilities connected to caregiving. Blyth et al. found that about one fifth of older caregivers reported pain. The level of pain experienced by a caregiver is a significant predictor of the overall caregiver burden [19] and increased mortality risk [17]; it can be assumed that pain also affects older caregivers’ HRQoL. The health effects of having pain and being an older caregiver were discussed by Blyth et al., who found that caregivers with pain reported more psychological stress and poorer health than caregivers without pain [7]. In the context of pain and HRQoL, the relations between being an older family caregiver, cognitive function, and health attitudes also need to be clarified. Thus, although HRQoL encompasses a narrower approach than QoL, i.e., health and health complaints, these factors should be controlled for, as they are all related to the aging process and QoL [20].

Gender must also be taken into consideration in studies on older caregivers, as pain is reported more frequently among elderly female caregivers than their male counterparts [21]. Kristensson Ekwall et al. [22] found that female caregivers were more vulnerable to the negative consequences of caregiving, as they found less satisfaction in care than males did and therefore might require greater consideration as family caregivers.

HRQoL is a concept that represents the ultimate goal of health promotion and is described as an individual cognitive appraisal of a person’s standard of living in relation to health [23]. HRQoL can be measured using instruments such as the Short-Form Health Survey, which includes dimensions of mental and physical health and is used worldwide. This should preferably be supplemented with other, more detailed measures of quality of life when used among older people, as it is short (fewer questions make it easier to answer) and does not include questions about work [24]. In literature, it has been stressed that the relations between mobility, functional ability, and HRQoL are important to consider in older people with pain [21], since pain may affect mobility and functional ability, as well as HRQoL.

In summary, older family caregivers may be subject to health problems of their own, due to advanced age and increased risk of health deterioration. Further, older family caregivers are largely responsible for the care of next-of-kin living at home, which can impact their physical and mental health both positively and negatively, due to effects on the day-to-day demands of life. Caregivers’ pain has not previously been studied in relation to HRQoL, although family caregivers can be expected to have pain to a large extent. Taking the above into account, we hypothesized that pain would be more common among family caregivers and have an impact on their QoL. Furthermore, the aim of this study was to investigate pain, HRQoL, and the associations of pain with HRQoL among older caregivers (aged 60 years and older), while controlling for demographic factors, functional and cognitive abilities, and health attitudes.

## Methods

### Study area and participants

This cross-sectional cohort study included individuals aged 60–96 years and was part of the Swedish National Study on Aging and Care (SNAC), an interdisciplinary, longitudinal, multi-center study initiated by the Swedish government and the Ministry of Social Affairs. The participants in the present study were recruited from SNAC-Blekinge (*n* = 1402), during 2001–2003, and from Good Aging in Skåne (GÅS) (*n* = 2931), during 2001–2004. The response rate was 60%.

The present study encompassed six municipalities in southern Sweden, covering rural areas, small towns, and a medium-sized town. The participants were randomly selected from a population register. The purpose of the SNAC design was to recruit a random sample with a broad age range, representing the population from 60 to 96 years, in 10 clusters. All surviving members of the 81-, 84-, 87-, 90-, 93-and 96-year-old cohorts were therefore invited. More details about the structure of the SNAC study have been presented by Lagergren et al. [25].

An invitation was sent by post to potential participants. If no response was received after 2 weeks, three attempts were made to contact the potential participants by phone. After providing signed informed consent, those who agreed to participate underwent medical examinations, cognitive tests, and functional assessments performed by the researchers, as well as completing a self-report questionnaire. The examination followed a predefined research protocol and took 3 h. The potential participants (*n* = 640; 444 women, 69.4%; 196 men, 30.6%; median age: 84 years, q_1_–q_3_ 78–90) who reported that they were both informal caregivers and received informal care themselves were excluded from this study. They were older than the study population on average, which could explain why they also received care. The potential participants with missing data for the question on whether they gave care were also excluded (*n* = 249; 147 women, 59%; 102 men, 41; median age: 84 years, q_1_–q_3_ 72–90).

Among the participants who reported being family caregivers (*n* = 379), 27 stated that they had been offered help or support within the preceding 3 months. In Sweden, where the participants lived, family caregiving is performed on a voluntary basis. According to the Swedish Social Services Act, a caregiving plan must be drawn up by the municipality’s officials and define, inter alia, 1) what actions are necessary; 2) the actions for which each principal is responsible; 3) which needs are supported by *someone other than* the municipality or county council; and 4) which principal has overall responsibility for the plan itself (SFS 2009:981, The Swedish Social Services Act).

The study was conducted in accordance with the Declaration of Helsinki (WMA, 2013). The ethics committee of Lund University (LU 128–00, LU 604–00, LU 744–00) approved the SNAC/GÅS study.

### Measurements and instruments

Data on the participants’ age, sex, education, housing, marital status, education, and financial situation were obtained from the SNAC self-report questionnaire (see Table 1). Functional ability was measured with four questions about laundry, shopping, food preparation, and housekeeping. Each item recorded the individual’s highest functional level (either 0 or 1) and was summed up to an index in accordance with Lawton and Brody’s algorithm of functioning (0–4, with 4 indicating independence) [26]. Mini-mental state estimation (MMSE), ranging from 0 to 30 (low values mean cognitive impairment), was used to assess individuals’ cognitive status [27] and was performed in interviews with the test leader.

**Table 1:** Demographic information on family caregivers and non-caregivers in the Swedish National Study on Aging and Care sample

	Total *n* = 3,444	Family caregivers *n* = 395	Non-caregivers *n* = 3,049	*p* value
**Demographic information**				
Age (years)				
Median, q_1_-q_3_	72, 66-81	66, 60-78	72, 66-81	**< 0.001**
Sex (female)	1,861/3,444 (54.0%)	212/395 (53.7%)	1,649/3,049 (54.1%)	< 0.887
Financial status (low)	535/3,324 (16.1%)	62/390 (15.9%)	473/2,934 (16.1%)	< 0.910
Education (low)				
Dropped out of school at 14–16 years old	2,414/2,770 (87.1%)	271/304 (89.1%)	2,143/2,466 (86.9%)	< 0.270
Married	1,895/3,390 (55.9%)	274/391 (70.1%)	1,621/2,999 (54.0%)	**< 0.001**
Living alone	1,399/3,394 (41.2%)	103/393 (26.2%)	1,296/3,001 (43.2%)	**< 0.001**
**Health variables**				
MMSE				
Median, q_1_-q_3_	28, 26-29	28, 26-29	28, 26-29	**< 0.001**
IADL (independence)	2,187/3,357 (65.1%)	266/389 (68.4%)	1,921/2,968 (64.7%)	**< 0.001**
Pain (yes)	1,853/3,303 (56.1%)	219/386 (56.7%)	1,634/2,917 (56.0%)	0.827
Pain intensity (VAS 1–10)				
Median, q_1_-q_3_	4, 3-5	4, 2-5	4, 3-5	**0.021**
VAS levels:				**0.023**
VAS 1.00–3.99	671/1,723 (38.9%)	101/215 (47.0%)	570/1,508 (37.8%)	
VAS 4.00–6.99	785/1,723 (45.6%)	83/215 (38.6%)	702/1,508 (46.6%)	
VAS 7.00–10.00	267/1,723 (15.5%)	31/215 (14.4%)	236/1,508 (15.6%)	
Pain location				
Neck	433/2,721 (15.9%)	55/339 (16.2%)	378/2,382 (15.9%)	0.874
Legs and feet	890/2,773 (32.1%)	98/342 (28.7%)	792/2,431 (32.6%)	0.155
Back	945/2,773 (34.1%)	107/344 (31.1%)	838/2,429 (34.5%)	0.224
Shoulder	691/2,773 (24.9%)	80/345 (23.2%)	611/2,428 (25.2%)	0.464
Joints	585/2,755 (21.2%)	59/345 (17.1%)	526/2,410 (21.8%)	**0.049**
Chest	144/2,703 (5.3%)	16/336 (4.8%)	128/2,365 (5.4%)	0.698
**Attitudes and quality of life**				
How soon I recover after illness depends on how I take care of myself (Entirely)	1,056/3,206 (32.9%)	120/383 (31.3%)	936/2,823 (33.2%)	0.108
I expect to have a very healthy life (Entirely)	475/3,191 (14.9%)	51/380 (13.4%)	424/2,811 (15.1%)	0.456
I am worried about my health:				0.327
Very worried	220/3,303 (6.7%)	24/381 (6.3%)	191/2,819 (6.8%)	
Fairly worried	807/3,303 (24.4%)	83/381 (21.8%)	703/2,819 (25.0%)	
A little worried	1,630/3,303 (49.3%)	205/381 (53.8%)	1,377/2,819 (48.8%)	
Not worried	646/3,303 (19.6%)	69/381 (18.1%)	548/2,819 (19.4%)	
**Mental HRQoL**				
MCS median, q_1_-q_3_	57.4, 51.9-59.9	57.1, 50.0-59.8	57.6, 52.0-60.0	0.064

Chi-squared tests were used for data at the nominal and ordinal levels, and the Mann Whitney U test was used for data at the interval levels. Significant factors are in bold

Information about having pain (yes/no), the location of pain, and the average pain intensity (one-dimensional Visual Analogue Scale [VAS], 1–10, a 10 cm continuum between the extremes of pain intensity: no pain and worst possible pain) was drawn from the SNAC protocol. The VAS is a reliable, validity-tested instrument [28] which can be used in a geriatric population [29]. In the analysis, VAS scores were divided into three levels (mild pain intensity: 1.00–3.99; moderate pain intensity: 4.00–6.99; severe pain intensity: 7.00–10.00); other studies have used other cut-off points, and no general cut-off points have been defined. A previous study analyzing the VAS scale found that a 3-class solution fit best [30]; therefore, this solution was used, along with the continuous VAS variable.

Attitudes toward health were examined through three items in the questionnaire: “How soon I recover after illness depends on how I take care of myself” [31], with the response options “a) Entirely” and “b) Not at all”; “I expect a very healthy life” [32], with the response options “a) Entirely” and “b) Not at all”; and “I am worried about my health” [32], with the response options “a) Very worried,” “b) Fairly worried,” “c) A little worried,” and “d) Not worried.”

HRQoL was measured using the self-report questionnaire Short-Form Health Survey (SF12), a generic instrument encompassing 12 questions. The instrument has been evaluated as a reliable and valid instrument for measuring HRQoL in the older population [33, 34]. The SF12 includes two components or dimensions of HRQoL: the mental component summary (MCS) and the physical component summary (PCS). Pain is included as an item in the physical dimension, so the PCS was not considered in the present study. The MCS includes six of the 12 items and four domains (role limitations, vitality, social function, and mental health). These are summed up in the MCS score used for the present study. The score ranges from 0 to 100, and higher numbers indicate higher HRQoL. Norm values for the MCS in the Swedish population, by age and sex, are available from Statistics Sweden [35].

### Statistical analysis

The study sample was analyzed in its entirety and divided into two groups: family caregivers and non-caregivers. Group-stratified analysis was also conducted. The dataset was large, but not normally distributed. For descriptive statistics, median and interquartile range (q_1_–q_3_) were used as continuous variables, while numbers and percentages (%) were used as categorical variables. To enable comparisons between groups (family caregivers vs. non-caregivers) to test differences in proportions, chi-squared tests were used for the nominal and ordinal data levels, and the Mann Whitney U test for the interval data levels.

The included variables were entered into logistic regression models for the total sample and stratified for caregiving to analyze their associations with mental HRQoL. Three logistic regression models (enter method) with three steps each were used. Step one included the pain variable. In step two, demographic variables were added, and in the last step, health variables were also added. Table 2, which presents the total sample, shows models in which the question concerning family caregiving was included in the first step. The score in the lowest quartile was defined as low mental HRQOL, with the cut-off of MCS = 51.88, and was used as the outcome variable, coded as 1. In the logistic regression analysis, all the variables were included, except for health attitudes, in which only one of three variables was included due to the risk of multicollinearity. The reference categories were younger age, being male, living with someone, independence in IADL, high financial resources, high education levels, low MMSE scores, being a non-caregiver, having no pain (VAS), and not being worried about one’s own health. The results were presented as odds ratios (ORs) with 95% confidence intervals (CIs). Collinearity diagnostics (variance inflation factors) were used to check for multicollinearity in the independent variables, and the factors were shown to be acceptable. Nagelkerke R squared was used to estimate the explained variation in the models. The Hosmer–Lemeshow goodness-of-fit test [36] was performed to determine whether the regression models fit the data. To test significance, a *p* value of < 0.05 was set. The statistical software SPSS version 24 for Windows (SPSS Inc., Chicago, IL, USA) was used for the analyses.

**Table 2:** Factors of importance to mental HRQoL in the total sample of SNAC-Blekinge and Good Aging in Skåne (GÅS)

	Model 1 (enter) (*n* = 2,459)			Model 2 (enter)			Model 3 (enter)		
	OR	CI	*p* value	OR	CI	*p* value	OR	CI	*p* value
Pain (VAS)	1.17	1.13–1.21	**< 0.001**	1.16	1.13–1.21	**< 0.001**	1.15	1.11–1.19	**< 0.001**
Family caregiver	1.23	0.92–1.64	0.154	1.38	1.03–1.85	**0.034**	1.47	1.08–1.99	**0.013**
Age				1.03	1.02–1.04	**< 0.001**	1.01	1.00–1.03	**0.011**
Gender				1.28	1.05–1.57	**0.016**	1.36	1.09–1.68	**0.005**
Living alone				1.21	0.97–1.50	0.086	1.17	0.93–1.46	0.173
Education				1.35	0.99–1.85	0.058	1.15	0.83–1.60	0.406
Financial status				1.93	1.51–2.48	**< 0.001**	1.79	1.38–2.32	**< 0.001**
Functional status (IADL)									0.105
Totally dependent									
Severely dependent									
Moderately dependent									
A little dependent									
MMSE							0.91	0.87–0.95	**< 0.001**
Worried about health									**< 0.001**
Very							4.48	2.94–6.83	**< 0.001**
Fairly							3.19	2.32–4.40	**< 0.001**
A little							1.60	1.18–2.17	**0.003**

Hosmer–Lemeshow test in model 1: *p* value = 0.602; model 2: *p* value = 0.602; model 3: *p* value = 0.371. Variables included in model 1: pain (VAS) and family caregiver. Nagelkerke R squared = 0.052. Variables included in model 2: pain (VAS), family caregiver, age, gender, living alone, education, and financial resources, Nagelkerke R squared = 0.105. Variables included in model 3: pain (VAS), family caregiver, age, gender, living alone, education, financial resources, functional status (IADL), MMSE, and worry about one’s own health, Nagelkerke R squared=0.172. The reference categories were the response alternatives: younger age, being male, living with someone, independence in IADL, high financial resources, high education levels, low MMSE scores, being a non-caregiver, having no pain (VAS), and not being worried about one’s own health. Significant factors are in bold

## Results

The median age in the sample was 72 years (q_1_–q_3_ 66–81) and 87.1% had a low education level (dropped out of school at age 14–16 years). Being a family caregiver was reported by 395 of 3444 (11.5%) participants, and 219 of the 386 (56.7%) caregivers reported pain. Those who were family caregivers were significantly younger, had higher cognitive ability (MMSE), were more frequently married or lived together with someone, and were less frequently dependent as regards functional ability (IADL) (*p* <  0.001). Caregivers and non-caregivers reported pain equally frequently, but non-caregivers more often reported high pain intensity (VAS 4, q_1_–q_3_ 2–5, vs. VAS 4, q_1_-q_3_ 3–5, *p* = 0.021). When the VAS score was divided into three levels, low pain intensity (VAS 1.00–3.99) was more commonly reported among caregivers (101/215, 47.0%) than among non-caregivers (570/1508, 37.8%, *p* = 0.023). Compared with non-caregivers (526/2410, 21.8%, *p* = 0.049), caregivers reported pain in the neck, legs and feet, back, and shoulders at the same rate, but less often reported pain in the joints (59/345, 17.1%). About 32% of the caregivers reported functional dependency. Irrespective of caregiver status, about one third of participants (28.1–31.8%) reported that they were very or fairly worried about their health. While mental HRQoL was lower among caregivers, the differences in proportions were not significant (*p* = 0.064) (Table 1).

In the total sample, pain (OR 1.15; CI 1.11–1.19) was associated with mental HRQoL when including the control variables (Table 2). The control variables were also associated with low mental HRQoL, with several of them showing a stronger association with HRQoL than pain intensity: being very worried about one’s own health (OR 4.48, CI 2.94–6.83), being fairly worried about one’s own health (OR 3.19, CI 2.32–4.40), being a little worried about one’s own health (OR 1.60, CI 1.18–2.17), financial resources (OR 1.79, CI 1.38–2.32), being female (OR 1.36, CI 1.09–1.68), age (OR 1.01, CI 1.00–1.03), cognitive impairment (MMSE) (OR 0.91, CI 0.87–0.95), and being a family caregiver (OR 1.47, CI 1.08–1.99). The variables included in the model explained 17.2% of the variance in mental HRQoL.

Among the family caregivers, pain was associated with low mental HRQoL (1.19, CI 1.07–1.32), which was not the case for any of demographic variables included – age, gender, education, or living conditions (Table 3). Furthermore, mental HRQoL was associated with being very worried about one’s own health (OR 10.83, CI 2.78–42.22), being fairly worried about one’s own health (OR 5.24, CI 1.89–14.57), financial resources (OR 2.68, CI 1.21–5.92), and cognitive ability (MMSE) (OR 0.83, CI 0.72–0.95). In the family caregiver group, the variance explained by the model was 22.8%.

**Table 3:** Factors of importance to mental HRQoL in family caregivers

	Model 1 (Backward LR) (*n* = 279)			Model 2 (enter)			Model 3 (enter)		
	OR	CI	*p* value	OR	CI	*p* value	OR	CI	*p* value
Pain (VAS)	1.21	1.10–1.33	**< 0.001**	1.22	1.11–1.35	**< 0.001**	1.19	1.07–1.32	**0.002**
Age				1.02	0.99–1.05	0.238	1.01	0.98–1.05	0.511
Gender				1.19	0.67–2.12	0.554	1.13	0.60–2.15	0.707
Living alone				0.85	0.44–1.64	0.619	0.61	0.30–1.26	0.181
Education				2.72	0.93–8.02	0.069	2.28	0.73–7.19	0.159
Financial status				2.07	1.01–4.24	**0.048**	2.68	1.21–5.92	**0.015**
Functional status (IADL)									0.595
Totally dependent									
Severely dependent									
Moderately dependent									
A little dependent									
MMSE							0.83	0.72–0.95	**0.007**
Worried about health									**0.002**
Very							10.83	2.78–42.22	**0.001**
Fairly							5.24	1.89–14.57	**0.001**
A little							2.42	0.96–6.09	0.060

Hosmer–Lemeshow test in model 1: *p* value = 0.965; model 2: *p* value = 0.445; model 3: *p* value = 0.793. Variables included in model 1: pain (VAS) and family caregiver. Nagelkerke R squared = 0.075. Variables included in model 2: pain (VAS), family caregiver, age, gender, living alone, education, and financial resources, Nagelkerke R squared = 0.12. Variables included in model 3: pain (VAS), family caregiver, age, gender, living alone, education, financial resources, functional status (IADL), MMSE, and worry about one’s own health, Nagelkerke R squared = 0.228. The reference categories were the response alternatives: younger age, being male, living with someone, independence in IADL, high financial resources, high education levels, low MMSE scores, having no pain (VAS), and not being worried about one’s own health. Significant factors are in bold

Also, among the non-caregivers, pain was associated with low mental HRQoL, but with slightly lower OR (1.15, CI 1.10–1.19) than among family caregivers (Table 4). In the non-caregiver group, HRQoL was associated with being very worried about one’s own health (OR 4.12, CI 2.63–6.45), being fairly worried about one’s own health (OR 3.06, CI 2.18–4.30), being a little worried about one’s own health (OR 1.52, CI 1.10–2.10), financial resources (OR 1.73, CI 1.31–2.29), being female (OR 1.40, CI 1.11–1.76), pain (OR 1.15, CI 1.10–1.19), age (OR 1.02, CI 1.00–1.03), and cognitive ability (MMSE) (OR 0.92, CI 0.88–0.95). This model explained 17% of the variance in mental HRQoL.

**Table 4:** Factors of importance to mental HRQoL in people who are not family caregivers

	Model 1 (Backward LR) (*n* = 2,180)			Model 2 (enter)			Model 3 (enter)		
	OR	CI	*p* value	OR	CI	*p* value	OR	CI	*p* value
Pain (VAS)	1.17	1.13–1.21	**< 0.001**	1.16	1.12–1.20	**< 0.001**	1.15	1.10–1.19	**< 0.001**
Age				1.03	1.02–1.04	**< 0.001**	1.02	1.00–1.03	**0.024**
Gender				1.30	1.05–1.62	**0.017**	1.40	1.11–1.76	**0.004**
Living alone				1.25	1.02–1.62	0.058	1.25	0.99–1.59	0.066
Education				1.25	0.90–1.73	0.184	1.07	0.76–1.52	0.688
Financial resources				1.76	1.35–2.30	**< 0.001**	1.73	1.31–2.29	**< 0.001**
Functional status (IADL)									0.064
Totally dependent									
Severely dependent									
Moderately dependent									
A little dependent									
MMSE							0.92	0.88–0.95	**< 0.001**
Worried about health									**< 0.001**
Very							4.12	2.63–6.45	**< 0.001**
Fairly							3.06	2.18–4.30	**< 0.001**
A little							1.52	1.10–2.10	**0.012**

Hosmer–Lemeshow test in model 1: *p* value = 0.282; model 2: *p* value = 0.608; model 3: *p* value = 0.074. Variables included in model 1: pain (VAS) and family caregiver. Nagelkerke R squared = 0.048. Variables included in model 2: pain (VAS), family caregiver, age, gender, living alone, education, and financial resources, Nagelkerke R squared = 0.105. Variables included in model 3: pain (VAS), family caregiver, age, gender, living alone, education, financial resources, functional status (IADL), MMSE, and worry about one’s own health, Nagelkerke R squared = 0.170. The reference categories were the response alternatives: younger age, being male, living with someone, independence in IADL, high financial resources, high education levels, low MMSE scores, having no pain (VAS), and not being worried about one’s own health. Significant factors are in bold

## Discussion

At the time of the study, 11.5% of the respondents were family caregivers, of whom 32% reported functional dependency and 56% reported pain. Being a caregiver was associated with low mental HRQoL. Caregivers reported pain to a similar extent as non-caregivers, but the pain intensity was lower among caregivers. The pain intensity likely had similar importance for HRQoL among caregivers and non-caregivers. Worry about one’s own health, low financial resources, and low cognitive status were also associated with low mental HRQoL in both groups, but with a higher OR among those giving care. These factors are all important to consider as clinical outcomes and when designing actions and goals in order to improve the health status of older family caregivers.

Although the pain intensity was lower among family caregivers, the prevalence of pain was similar for family caregivers and non-caregivers. The pain prevalence among caregivers in this study was in line with the findings for the whole sample in SNAC-Blekinge, with 769 of 1402 (54.9%) participants reporting pain [1], and with the findings of Rottenberg et al. [2], with pain at ≥2 sites reported by 42.3% of participants at the age of 70 years and by 54.6% at 78 years. Blyth et al. found a lower prevalence of pain (chronic pain: 19.2% in men, 23.3% in women) among caregivers [7] than that found in the present study (pain in preceding 4 weeks: 56.7%). However, these three studies used different questions regarding pain frequency, which may have affected the results and needs to be considered when comparing the different proportions.

In line with the results of the present study, caring for someone at home has previously been found to be associated with bodily pain [2]. Having pain and being a family caregiver both have negative health impacts [7, 37]. However, direct comparisons with results from other studies might be prevented by the lack of a comparative methodology to apply to family caregivers and non-caregivers. One could argue that one explanation for the result of lower pain intensity among family caregivers could be the significantly lower age of the family caregivers. Although the median age differed by 6 years between caregivers and non-caregivers, the caregivers likely had the same pain conditions as their older counterparts. This requires attention as caregivers grow older and their bodily pain may increase. However, a sub-analysis performed in the sample (not presented) showed that age had an association with pain intensity, but not with pain frequency, leading to uncertainty about the relation between age and pain. Another possible explanation is that people included in the group of non-caregivers were individuals who had health complaints and comorbidity to a higher degree, i.e., they had refrained from providing care and support to family members due to their own poor health status. A finding that supports this explanation is the comparison between the caregivers and non-caregivers as regards pain locations. The non-caregivers more often reported pain in joints. They also reported higher pain intensity and dependency in IADL compared with the caregivers. To the best of the authors’ knowledge, this paper and the study by Del Río Lozano et al. [38] are the first attempts to compare pain and HRQoL between caregivers and non-caregivers. Del Río Lozano et al. found gender differences, with a poorer situation and more discomfort or pain among women [38]. More studies are needed to understand the pain progression and how it impacts on HRQoL over time among those in old age who are and are not family caregivers.

A crucial finding for highlighting the caregivers’ health situation was that they reported low HRQoL to the same degree as the non-caregivers did, even though the caregivers reported lower pain intensity, less pain, and more often were independent in activities in daily living. This indicates that it is important to place the living situation of older family caregivers in a broader context. For instance, the results suggest that worry about one’s own health and low financial resources were equally common among caregivers and non-caregivers (31 and 16% in the total sample, respectively) and were associated with low mental HRQoL in both groups, but more strongly among family caregivers. Maryam et al. suggested that vulnerable caregivers, who represented one fourth of caregivers, were more likely to be over 65 years old, to have difficulty providing care, and to report that their own health had been a problem in giving care [37]. The majority (87.1%) in the sample had a rather low education level and likely had limited health literacy, affecting performance of health-related duties. A negative feeling of being dependent on others can impact on HRQoL. For example, caregivers who need help – and especially those who need to ask for it – have reported lower QoL than those who do not [39].

The strong association between worry about one’s own health and HRQoL could be explained by the caregivers’ feeling of responsibility and fear of negative consequences in their absence. Such a self-imposed duty could affect them negatively, even though being a caregiver was voluntary in this study population, in accordance with the Swedish Social Services Act. Schulz et al. discussed that stress and physical strain, in addition to a lack of choice in becoming a family caregiver, increase caregivers’ worry and anxiety [8]. Acton found that health-promoting self-care behavior could act as a mediator decreasing the effect of caregiver stress on HRQoL [40].

In a previous study by Alejandro and colleagues, they discussed that the social and familial expectations to step up and care for loved ones could increase the risk of low HRQoL, negative health outcomes, and poor financial situations among family caregivers [41]. As the present study indicates, Savage and Bailey suggested the importance of caregivers’ financial situation for HRQoL [42]. Those taking care of their loved ones have been called ‘the unsung heroes’ of society [17], as research has suggested that socio-economic inequalities in morbidity by income disappear in old age [43] and that pain increases mortality risk. Ostward mentioned that caregivers sometimes neglect their own health to provide care to their diseased relatives, thinking that they are not entitled to make time for themselves [44]. Addressing caregivers’ worries requires understanding of the individuals’ own goals and expectations, as well as of the caregiver role.

It is noteworthy that the model could only explain a part of the variance of HRQoL (22.8%), even though we included factors expected to be important for caregivers’ HRQoL. Worries about one’s own health, financial resources, and cognitive ability, rather than pain, were behind the variance. For that reason, other individual and environmental factors need to be considered in future research. Other factors related to HRQoL include social activities and leisure activities, which could also be compromised in the caregiving situation. More attention should also be paid to the situation of aging family caregivers who have increased risks of poor physical health, functional problems, and not being taken care of at an early age, especially given the increasing number of caregivers.

The strength of this study was the inclusion of a large, representative study sample with a broad age range. The size of the study sample was important, as the family caregiver group represented more than one tenth of the total study population. Pain was measured by whether the participants reported pain during the preceding 4 weeks. The strength of this question was that it has been used in other studies on pain in older adults, including in Europe [45]. Furthermore, to control for unique and caregiver-specific variations in pain on mental HRQoL, separate models were used for family caregivers and non-caregivers. Although this entailed a risk of losing variation in the dependent variable (HRQoL), a logistic regression was performed. The choice was based on the fact that the items in the SF12 represented a non-parametric level and the interest was in finding the characteristics of those with low mental HRQoL.

The external and internal dropouts were older, which may have skewed the sample towards a healthier group than the overall population and may be a threat to validity. To compensate for increased risk of external dropouts due to advanced age, oversampling was performed in the oldest age groups. This sampling strategy resulted in an age distribution roughly similar to that in the municipalities and in Sweden in general [46]. Furthermore, the sample fully reflected the aging population in age and gender. Additionally, proxies and home visits were used to reduce internal dropout. It should be noted that the Swedish Social Services Act means that caregiving is the responsibility of municipalities. Although acting as a caregiver is voluntary in Sweden, family caregivers currently provide more care than the public health care system [15]. The majority of the participants were non-caregivers, which may have affected the results. However, as the frequencies were reported in percentage points and the statistical calculations in SPSS took into account the distributions between the groups, the risk of this was considered minimal. The cross-sectional design used in this study limited the causal inferences from the results.

## Conclusion

This study suggested that the prevalence rates of pain were similar among family caregivers and non-caregivers, as was the association of pain with mental HRQoL. However, pain intensity was lower among family caregivers. This study also showed that other factors were of importance for HRQoL in older family caregivers. Worry about one’s own health and low financial resources were common and associated with mental HRQoL in both groups, but with a higher odds ratio among caregivers.

## Data Availability

All data analyzed during this study are included in this published article.

## Abbreviations

GÅS: Good Aging in Skåne
HRQoL: Health-related quality of life
IADL: Instrumental activities of daily living
MCS: Mental component summary
MMSE: Mini-mental state estimation
SF12: Short Form Health Survey 12
SNAC–Blekinge: Swedish National Study on Aging Care in Blekinge
VAS: Visual analogue scale
